# A Retrospective Assessment of Guideline Adherence and Treatment Outcomes From *Clostridioides difficile* Infection Following the IDSA 2021 Clinical Guideline Update: *Clostridioides difficile* Infection

**DOI:** 10.1093/ofid/ofae524

**Published:** 2024-09-30

**Authors:** Erik R Dubberke, Qinghua Li, Engels N Obi, Vladimir Turzhitsky, Fakhar Siddiqui, Brian H Nathanson

**Affiliations:** Washington University School of Medicine, Saint Louis, Missouri, USA; Merck & Co., Inc., Rahway, New Jersey, USA; Merck & Co., Inc., Rahway, New Jersey, USA; Merck & Co., Inc., Rahway, New Jersey, USA; Merck & Co., Inc., Rahway, New Jersey, USA; OptiStatim, LLC, Longmeadow, Massachusetts, USA

**Keywords:** 2021 clinical guideline update, *Clostridioides difficile*, fidaxomicin, metronidazole, vancomycin

## Abstract

**Background:**

The 2021 update to the Infectious Diseases Society of America *Clostridioides difficile* infection (CDI) guidelines recommended fidaxomicin as the preferred treatment over vancomycin for patients with initial and recurrent CDI. Few studies have examined how treatment patterns and clinical outcomes of hospitalized CDI patients changed after the postguideline update or contemporary real-world outcomes of fidaxomicin vs vancomycin.

**Methods:**

This retrospective, observational study used the PINC AI Healthcare Database on adult patients who received CDI treatment between 1/2020 and 6/2021 (pre period) and between 10/2021 and 9/2022 (post period). We examined treatment patterns of fidaxomicin, vancomycin, and metronidazole, as well as clinical and health care resource use outcomes of patients treated exclusively with fidaxomicin vs vancomycin, using nearest-neighbor propensity matching and hierarchical regression methods. As a sensitivity analysis, we repeated the fidaxomicin vs vancomycin comparisons among patients with recurrent and nonrecurrent index infections.

**Results:**

A total of 45 049 patients with CDI from 779 US hospitals met initial inclusion criteria. Comparing the pre vs post periods, the proportion of patients treated with fidaxomicin increased from 5.9% to 13.7% (*P* < .001), vancomycin use decreased from 87.9% to 82.9% (*P* < .001), and metronidazole use decreased from 21.6% to 17.2% (*P* < .001). When comparing fidaxomicin vs vancomycin in the post period, fidaxomicin was associated with lower CDI recurrence (6.1% vs 10.2%; *P* < .001) and higher sustained clinical response (91.7% vs 87.8%; *P* < .001). Ninety-day postdischarge costs were not significantly different between groups. A sensitivity analyses showed similar findings.

**Conclusions:**

Since the 2021 guideline update, fidaxomicin use has increased significantly but could be further utilized given its association with better clinical outcomes and no increase in postdischarge costs.

*Clostridioides difficile* is a gram-positive, spore-forming anaerobic bacterium. The negative impact of a *Clostridioides difficile* infection (CDI) is considerable. CDI affects ∼460 000 patients in the United States and is the most common health care–associated infection in adults [1–4]. CDI is also the number one cause of nosocomial diarrhea in the United States. Recurrent infections are common, with ∼1 in 6 patients having a recurrent CDI infection within 8 weeks [1]. Furthermore, Lessa et al. reported a 30-day crude case fatality rate of 9.3% for patients with health care–associated CDI [5]. US inpatient costs of CDI are ∼$5 billion annually, and Zhang et al. reported health care costs attributable to primary CDI over a 6-month follow-up period to be ∼$24 000 per patient [6–8].

Historically, vancomycin and metronidazole were first-line treatments for CDI, with vancomycin recommended for more severe cases [9]. However, treatment of CDI evolved as the antibiotic fidaxomicin received approval by the US Food and Drug Administration in 2011 based on the results of its phase 3 trials [2, 9–11]. Fidaxomicin has bactericidal activity against *C. difficile* but less activity than metronidazole and vancomycin against commensal bacterial species in the gut that suppress colonization of *C. difficile* [12–16]. Importantly, the Infectious Diseases Society of America (IDSA) and Society for Healthcare Epidemiology of America (SHEA) guidelines for the treatment of CDI have changed within the last 6 years. In 2017, metronidazole was demoted from a first-line agent for nonsevere CDI [2, 4]. In 2021, the guidelines were further updated to have fidaxomicin as the only recommended first-line agent for CDI due to its superior sustained clinical response, with vancomycin as an alternative agent [2, 4, 17].

There is an evidence gap on how CDI treatment patterns changed after the 2021 clinical guideline updates nationally. There is some evidence that fidaxomicin use has been limited, presumably as it is more expensive than vancomycin [18]. Dubberke et al. examined how guideline changes since 2021 have impacted treatment of CDI in Medicare outpatients [2], but a large, multicenter study examining treatment patterns of all adult patients hospitalized with CDI is lacking. Moreover, comparative clinical outcomes between fidaxomicin- and vancomycin-treated patients with CDI are unknown in a contemporary population. This study addresses these critical gaps in the literature.

## METHODS

This was a retrospective, observational study using the PINC AI Healthcare Database, an electronic laboratory, pharmacy, and billing data repository representing ∼25% of US hospitals. The analysis was conducted in 2 parts. First, we compared the use of fidaxomicin, vancomycin, and metronidazole pre– vs post–IDSA 2021 guidelines in adults hospitalized with CDI. Next, we compared clinical and economic outcomes during the study period in patients with CDI treated exclusively with fidaxomicin or vancomycin.

### Sample Selection for Pre– vs Post–IDSA 2021 Guideline Analyses

We examined all hospitalized inpatients aged 18 and older with an International Classification of Diseases, 10th Revision (ICD-10), code for CDI (A04.7x). Patients were required to have received oral vancomycin, oral fidaxomicin, oral or intravenous (IV) metronidazole for at least 10 consecutive days during hospitalization or would be eligible for study inclusion provided that they received vancomycin, fidaxomicin, or metronidazole on their last day of hospitalization. Patients missing key demographic or outcome data were excluded. We stratified the data by pre- vs postpublication of the IDSA 2021 guideline updates. We defined the pre period of treatment to be patients admitted between 1/2020 and 6/2021. The post period was defined as patients admitted between 10/2021 and 9/2022 (the latest data available at the time of analysis). Patients admitted between 7/2021 and 9/2021 (ie, the first 3 months postpublication of the updated guidelines) were in the “washout” period and were excluded from the analysis. Only the first CDI admission was analyzed among patients with multiple admissions, and patients without at least 1 record in the PINC AI Healthcare Database within 365 days before the index admission were excluded to help ensure that we correctly classified the index CDI admission and accurately captured baseline characteristics.

### Sample Selection for Fidaxomicin vs Vancomycin Subgroup Analyses

For the second analysis, we created a pooled subset of pre and post period patients from the first analysis who were treated exclusively with fidaxomicin (ie, without any treatment with vancomycin or metronidazole) or treated with vancomycin (ie, without any treatment with fidaxomicin or metronidazole). Patients in this analysis were categorized as having nonrecurrent initial CDI or recurrent CDI based on the following criteria: Nonrecurrent initial CDI was defined as a diagnosis of nonrecurrent CDI (ICD-10-CM code = A04.72) on the index hospital admission record and no CDI diagnosis within 3 months (90 days) before the index CDI infection episode. Recurrent CDI was defined as a diagnosis of recurrent CDI (ICD-10-CM code = A04.71) on the index hospital admission record or a CDI diagnosis (ICD-10-CM code = A04.71 or A04.72) on both the index hospital admission record and any record within 3 months (90 days) before the index hospital admission date. Clinical outcomes including clinical resolution, sustained clinical response, and CDI recurrence were defined as consistently with the literature as possible (Supplementary Table 1) given the data available in the PINC AI Healthcare Database. CDI-related readmissions or emergency room visits were defined by the presence of an ICD-10 code of A04.7× either as a primary or comorbid condition.

### Statistical Analyses

To describe the variables in the data set, we derived the mean, SD, median, and interquartile range for continuous variables and counts and frequencies for categorical variables. Patient costs were inflation-adjusted to the last half of 2022 based on medical costs from the Consumer Price Index [19]. Patient costs derived at 30, 60, and 90 days postdischarge excluded survivors who were discharged to a hospice location. Comparisons between groups were done using the Student *t* test for continuous variables and the chi-square test for categorical variables.

### Pre– vs Post–IDSA 2021 Guideline Analyses

For this analysis, the focus was on patients who received fidaxomicin, vancomycin, or metronidazole for 10 consecutive days during hospitalization and/or on the last day of hospitalization. To describe rates of fidaxomicin use at the hospital level, we restricted the analysis to hospitals contributing at least 25 study-eligible patients.

We then derived a multilevel logistic regression model with hospital-level random effects for all study-eligible patients with the dependent variable being fidaxomicin use at the patient level. The key predictors of interest were the hospital characteristics of census region, urbanicity, size (ie, number of beds), and teaching status, with an indicator variable of pre vs post period as a main effect and effect modifier for the hospital characteristics. Covariates used in the regression model included patient age, gender, Charlson Comorbidity Index, intensive care unit (ICU) admission, mechanical ventilation, dialysis, H2 blockers, proton pump inhibitors, and high/low-risk antibiotics present on or before the first day of treatment for CDI (Supplementary Table 2). We then calculated marginal effects for these variables after multivariable adjustment, along with residual intraclass correlation to measure the proportion of the residual variance explained by individual hospitals.

### Fidaxomicin vs Vancomycin Subgroup Analyses

A propensity matching approach was used to compare clinical and economic outcomes between fidaxomicin and vancomycin. The propensity score model was created using a logistic regression model with receipt of fidaxomicin (vs vancomycin) as the dependent variable, and independent variables were all variables presented in Supplementary Table 3. As we wanted to examine outcomes based on either all patients (eg, in-hospital mortality), survivors only (eg, 30-day readmission), or survivors not discharged to hospice (eg, 30-day total costs postdischarge), we derived 3 separate propensity scores on populations eligible for the outcomes of interest. We then repeated this analysis separately for both nonrecurrent and recurrent CDI patients. Propensity matching was done using a 1:1 nearest neighbor algorithm with a caliper of 0.25 times the standard deviation of the propensity score. We assessed covariate balancing by calculating Cohen's *d* statistic and considered a standardized difference >0.1 to indicate an imbalance. After propensity matching, inference tests comparing fidaxomicin vs vancomycin took the paired nature of the data into account on the matched samples [20]. As the continuous outcomes of costs and length of stay were skewed, we assumed the outcomes followed a gamma distribution and used a logarithmic link function to derive confidence intervals and *P* values.

As a sensitivity analysis, we derived the mean marginal effects of fidaxomicin vs vancomycin using multilevel hierarchical models with hospitals as random effects based on all eligible patients (as opposed to inferences derived from propensity-matched samples). For binary outcomes, we used multilevel mixed-effects logistic regression. For continuous outcomes (eg, postindex length of stay), we used multilevel generalized linear models that assumed a gamma distribution and logarithmic link function. For the continuous cost outcomes postdischarge, we observed that a disproportionate number of patients had 0 costs, and in these cases, we used a 0-inflated Poisson regression approach with robust clustering at the hospital level [20].

We considered *P* values <.05 to be statistically significant. All analyses were done using Stata/MP 18.0 for Windows (StataCorp, LLC, College Station, TX, USA). Because this study utilized already existing Health Insurance Portability and Accountability Act (HIPAA)–compliant, fully de-identified data, it was exempt from institutional review board review [21].

## RESULTS

A total of 45 049 patients from 779 hospitals met all study inclusion criteria for the first analysis (Supplementary Table 4). We observed that 85.9% (38 713/45 049) of patients had nonrecurrent CDI on their index admission (Supplementary Table 3). There were 29 520 patients in the pre period (65.5% of the cohort) and 15 529 patients in the post period. In the pre period, the mean (SD) age was 68.8 (15.9) years, 41% were male, and 80% were Caucasian. Similarly, in the post period, the mean (SD) age was 67.5 (15.6) years, 41% were male, and 81% were Caucasian. In the pre period, 10.6% were in the ICU on their first day of treatment (index day) vs 9.7% in the post period (*P* = .005). The in-hospital mortality rate was 3.94% in the pre period vs 4.58% in the post period (*P* = .001). However, the percentages of patients dying in-hospital or discharged to hospice were almost identical in the pre period vs post period: 8.49% vs 8.45% (*P* = .884). Among all survivors, there were fewer all-cause and CDI-related readmissions within 90 days postdischarge in the post vs pre period. Differences in chronic comorbidities, hospital interventions, and other outcomes between patients in the post vs pre period tended to be statistically nonsignificant, with some outcomes being significant but clinically modest (Supplementary Table 5).

**Figure 1.**
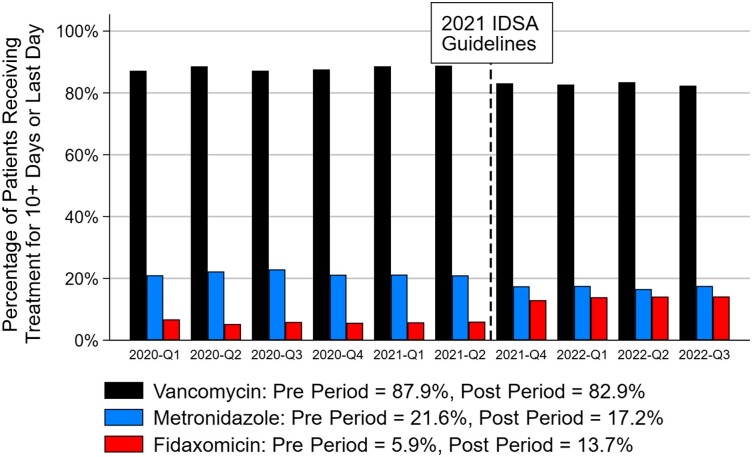
The quarterly *Clostridioides difficile* infection treatment utilization pattern of vancomycin, metronidazole, and fidaxomicin pre– vs post–IDSA 2021 guideline update. Figure 1 shows the percentage of patients receiving vancomycin, metronidazole, or fidaxomicin before vs after the IDSA 2021 guideline update by quarter. Note that the treatments at the patient level were not mutually exclusive. Abbreviations: IDSA, Infectious Diseases Society of America; Q, quarterly.

**Figure 2.**
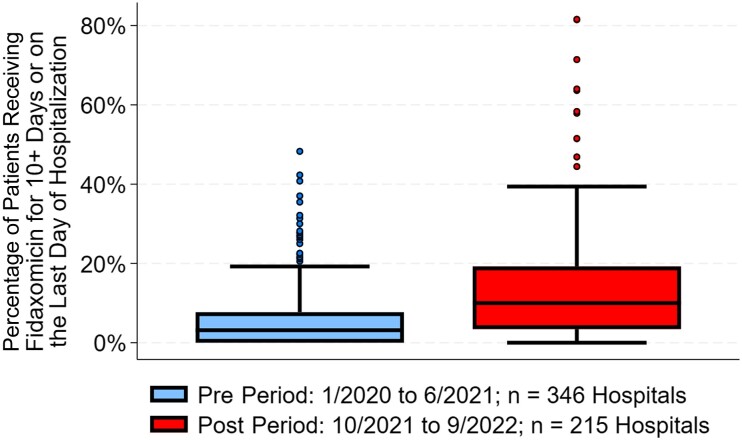
Box plots of the percentage of *Clostridioides difficile* infection patients treated with fidaxomicin at the hospital level pre– vs post–IDSA 2021 guideline update. Abbreviation: IDSA, Infectious Diseases Society of America.

Figure 1 shows the treatment patterns of vancomycin, metronidazole, and fidaxomicin use by quarter for all patients in the pre and post periods. We observed a decrease from the pre period to the post period for both vancomycin (87.9% [25 956/29 520] to 82.9% [12 877/15 529]; *P* < .001) and metronidazole (21.6% vs 17.2%; *P* < .001). In contrast, fidaxomicin use increased pre vs post period (5.9% vs 13.7%; *P* < .001), but it was still the least used treatment in the post period. There were 359 hospitals in total that treated 25 or more study-eligible CDI patients. At the hospital level, the median (IQR) fidaxomicin use rate was 3.1% (0%–7.7%) in the pre period and 10.0% (3.4%–19.2%) in the post period (*P* < .001) (Figure 2).

**Figure 3.**
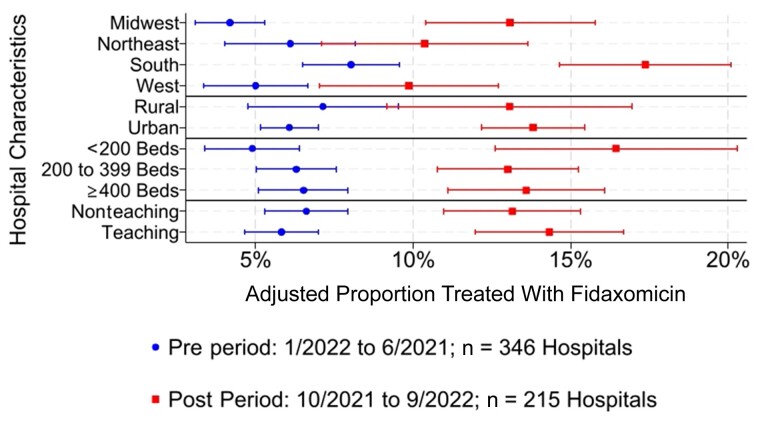
Adjusted marginal effects with 95% confidence intervals of fidaxomicin use pre– vs post–IDSA 2021 guideline update. Abbreviation: IDSA, Infectious Diseases Society of America.

From the hierarchical model, we observed that the pre vs post period significantly modified the effect of each hospital characteristic variable (region, urbanicity, size, teaching status). Marginal effects with 95% CIs are depicted in Figure 3, and it is notable how the changes from pre vs post period varied across hospital characteristics. For example, increases in fidaxomicin use were the smallest in the northeast and greatest in the south among the census regions (Figure 3). The residual intraclass correlation was 31.2% (95% CI, 27.0%–35.8%), which indicates that 31.2% of the variance in fidaxomicin use not explained by the patient and hospital characteristics included in the model is attributed to differences between hospitals (*P* < .001).

For the second analysis comparing clinical and economic outcomes between fidaxomicin and vancomycin, 24 853 patients met inclusion criteria (Supplementary Table 4), with 1075 (4.3%) treated with fidaxomicin. Supplementary Table 6 shows the number of matched pairs for each data set and for each set of applicable outcomes after 1:1 nearest neighbor propensity matching. In each propensity-matched set, all covariates in the propensity score were adequately balanced. Table 1 shows the propensity-matched clinical outcomes for the overall cohort and for the nonrecurrent and recurrent CDI patients. Table 2 shows the propensity-matched economic outcomes for survivors not discharged to hospice for the same 3 groups.

**Table 1.** Clinical Outcome Results for Fidaxomicin vs Vancomycin Comparison After 1:1 Nearest Neighbor Propensity Matching

	Vancomycin, Rate or Mean (95% CI)	Fidaxomicin, Rate or Mean (95% CI)	*P* Value
Overall Cohort			
Clinical resolution	98.12% (97.30%–98.93%)	98.02% (97.18%–98.86%)	.876
Sustained clinical response	88.23% (86.29%–90.17%)	91.71% (90.05%–93.37%)	**.008**
CDI recurrence	10.08% (8.25%–11.91%)	6.44% (4.94%–7.93%)	**.003**
In-hospital mortality	1.88% (1.07%–2.70%)	2.17% (1.29%–3.04%)	.640
In-hospital mortality or discharged to hospice	4.52% (3.27%–5.77%)	5.08% (3.76%–6.41%)	.545
Total length of stay	7.66 (6.96–8.37)	7.62 (7.13–8.11)	.927
All-cause readmission within 30 d	20.49% (18.02%–22.95%)	17.28% (14.97%–19.59%)	.062
All-cause readmission within 60 d	30.19% (27.39%–33.00%)	26.89% (24.18%–29.60%)	.094
All-cause readmission within 90 d	34.17% (31.28%–37.07%)	31.36% (28.52%–34.19%)	.164
CDI-related readmission within 30 d	9.71% (7.90%–11.52%)	6.60% (5.08%–8.12%)	**.012**
CDI-related readmission within 60 d	12.72% (10.68%–14.75%)	9.13% (7.37%–10.89%)	**.008**
CDI-related readmission within 90 d	13.98% (11.86%–16.10%)	10.97% (9.06%–12.88%)	**.034**
ER visits within 30 d	11.84% (9.87%–13.82%)	9.32% (7.54%–11.10%)	.060
ER visits within 60 d	17.77% (15.43%–20.10%)	14.56% (12.41%–16.72%)	**.048**
ER visits within 90 d	20.87% (18.39%–23.36%)	17.38% (15.06%–19.69%)	**.044**
CDI-related ER visits within 30 d	1.46% (0.72%–2.19%)	1.17% (0.51%–1.82%)	.565
CDI-related ER visits within 60 d	1.84% (1.02%–2.67%)	1.75% (0.95%–2.55%)	.869
CDI-related ER visits within 90 d	1.84% (1.02%–2.67%)	2.04% (1.18%–2.90%)	.752
Total hospital costs	$19 765 ($17 598–$21 933)	$21 452 ($19 402–$23 503)	.273
Postindex LOS	5.18 (4.69–5.67)	5.21 (4.89–5.54)	.900
Hospital costs postindex	$12 001 ($10 788–$13 213)	$13 305 ($12 184–$14 425)	.128
Nonrecurrent CDI Cohort			
Clinical resolution	98.56% (97.63%–99.49%)	97.60% (96.40%–98.80%)	.226
Sustained clinical response	87.84% (85.28%–90.40%)	91.84% (89.69%–93.99%)	**.020**
CDI recurrence	10.88% (8.42%–13.34%)	5.90% (4.03%–7.77%)	**.002**
In-hospital mortality	1.44% (0.51%–2.37%)	3.36% (1.95%–4.77%)	**.033**
In-hospital mortality or discharged to hospice	4.96% (3.26%–6.66%)	6.24% (4.34%–8.14%)	.326
Total length of stay	8.73 (7.71–9.74)	8.80 (8.09–9.51)	.905
All-cause readmission within 30 d	21.00% (17.74%–24.26%)	17.00% (13.99%–20.01%)	.066
All-cause readmission within 60 d	29.33% (25.69%–32.98%)	26.50% (22.97%–30.03%)	.262
All-cause readmission within 90 d	32.33% (28.59%–36.08%)	30.83% (27.14%–34.53%)	.570
CDI-related readmission within 30 d	7.67% (5.54%–9.80%)	5.67% (3.82%–7.52%)	.170
CDI-related readmission within 60 d	10.17% (7.75%–12.59%)	7.50% (5.39%–9.61%)	.100
CDI-related readmission within 90 d	11.17% (8.64%–13.69%)	9.33% (7.00%–11.66%)	.284
ER visits within 30 d	11.33% (8.79%–13.87%)	10.17% (7.75%–12.59%)	.518
ER visits within 60 d	18.50% (15.39%–21.61%)	14.83% (11.99%–17.68%)	.079
ER visits within 90 d	22.00% (18.68%–25.32%)	16.67% (13.68%–19.65%)	**.017**
CDI-related ER visits within 30 d	1.33% (0.41%–2.25%)	1.00% (0.20%–1.80%)	.595
CDI-related ER visits within 60 d	1.83% (0.76%–2.91%)	1.33% (0.41%–2.25%)	.493
CDI-related ER visits within 90 d	1.83% (0.76%–2.91%)	1.50% (0.53%–2.47%)	.638
Total hospital costs	$23 437 ($20 091–$26 782)	$25 884 ($22 758–$29 009)	.241
Postindex LOS	5.47 (4.81–6.12)	5.56 (5.13–6.00)	.809
Hospital costs postindex	$13 014 ($11 267–$14 761)	$14 717 ($13 210–$16 223)	.136
Recurrent CDI Cohort			
Clinical resolution	98.34% (97.12%–99.56%)	98.58% (97.45%–99.71%)	.782
Sustained clinical response	86.49% (83.23%–89.76%)	92.42% (89.89%–94.95%)	**.005**
CDI recurrence	12.05% (8.91%–15.18%)	6.25% (3.92%–8.58%)	**.004**
In-hospital mortality	1.18% (0.15%–2.22%)	0.47% (−0.18%–1.13%)	.274
In-hospital mortality or discharged to hospice	4.03% (2.15%–5.91%)	3.32% (1.61%–5.03%)	.578
Total length of stay	6.46 (5.74–7.18)	6.02 (5.43–6.62)	.361
All-cause readmission within 30 d	18.85% (15.10%–22.60%)	16.95% (13.35%–20.54%)	.487
All-cause readmission within 60 d	27.68% (23.40%–31.97%)	27.21% (22.94%–31.47%)	.880
All-cause readmission within 90 d	32.70% (28.20%–37.19%)	31.98% (27.51%–36.45%)	.830
CDI-related readmission within 30 d	8.83% (6.11%–11.55%)	7.40% (4.89%–9.91%)	.454
CDI-related readmission within 60 d	13.13% (9.89%–16.36%)	11.46% (8.40%–14.51%)	.478
CDI-related readmission within 90 d	15.04% (11.61%–18.46%)	13.37% (10.10%–16.63%)	.507
ER visits within 30 d	12.17% (9.04%–15.31%)	8.59% (5.91%–11.28%)	.093
ER visits within 60 d	19.09% (15.33%–22.86%)	14.80% (11.39%–18.20%)	.110
ER visits within 90 d	23.63% (19.56%–27.70%)	19.09% (15.33%–22.86%)	.110
CDI-related ER visits within 30 d	2.86% (1.27%–4.46%)	1.43% (0.29%–2.57%)	.166
CDI-related ER visits within 60 d	3.10% (1.44%–4.76%)	2.39% (0.92%–3.85%)	.533
CDI-related ER visits within 90 d	3.34% (1.62%–5.06%)	2.86% (1.27%–4.46%)	.695
Total hospital costs	$15 839 ($13 790–$17 889)	$15 442 ($13 305–$17 578)	.791
Postindex LOS	5.17 (4.55–5.79)	4.80 (4.31–5.29)	.361
Hospital costs postindex	$12 084 ($10 187–$13 980)	$11 587 ($9886–$13 289)	.706

Sample sizes for the various outcomes are provided in Supplementary Table 6.

Boldface *P* values are < .05.

Abbreviations: CDI, *Clostridioides difficile* infection; ER, emergency room; LOS, length of stay.

**Table 2.** Economic Outcome Results for Fidaxomicin vs Vancomycin Comparison After 1:1 Nearest Neighbor Propensity Matching

	Vancomycin, Mean (95% CI)	Fidaxomicin, Mean (95% CI)	*P* Value
Overall Cohort			
All costs postdischarge within 30 d for survivors	$6266 ($5002–$7530)	$5075 ($4256–$5894)	.113
All costs postdischarge within 60 d for survivors	$9404 ($7898–$10 910)	$8737 ($7592–$9882)	.487
All costs postdischarge within 90 d for survivors	$12 875 ($10 968–$14 782)	$11 055 ($9729–$12 382)	.115
Nonrecurrent Cohort			
All costs postdischarge within 30 d for survivors	$6096 ($4018–$8174)	$5453 ($4288–$6619)	.584
All costs postdischarge within 60 d for survivors	$8657 ($6468–$10 847)	$9460 ($7777–$11 144)	.562
All costs postdischarge within 90 d for survivors	$11 154 ($8784–$13 524)	$11 735 ($9843–$13 626)	.703
Recurrent Cohort			
All costs postdischarge within 30 d for survivors	$4828 ($3790–$5865)	$4279 ($3206–$5351)	.465
All costs postdischarge within 60 d for survivors	$10 655 ($7199–$14 110)	$7356 ($5956–$8755)	.055
All costs postdischarge within 90 d for survivors	$13 773 ($9704–$17 843)	$9830 ($8052–$11 608)	.059

From Table 1, we observed that fidaxomicin was associated with a higher sustained clinical response rate than vancomycin in the overall cohort (91.71%; 95% CI, 90.05%–93.37%; vs 88.23%; 95% CI, 86.29%–90.17%; *P* = .008). With vancomycin as the reference category, this translates into an odds ratio (OR) of 1.48 (95% CI, 1.11–1.97) and a number needed to treat of 29 (ie, on average, 29 patients would have to receive fidaxomicin for 1 additional patient to have a sustained clinical response). Fidaxomicin also had a lower CDI recurrence rate (6.44%; 95% CI, 4.94%–7.93%; vs 10.08%; 95% CI, 8.25%–11.91%; *P* = .003). This translates to an OR of 0.61 (95% CI, 0.44–0.85) and a number needed to treat of 28 (ie, on average, 28 patients would need to receive fidaxomicin for 1 additional patient to not have a CDI recurrence). Similar statistically significant findings in favor of fidaxomicin over vancomycin were observed for these 2 clinical outcomes within the nonrecurrent and recurrent CDI subsets (Table 1). Fidaxomicin was also associated with fewer CDI-related readmissions in the overall cohort at 30, 60, and 90 days compared with vancomycin, as well as fewer all-cause ER visits at 60 and 90 days. These findings were similar but generally did not reach statistical significance in the nonrecurrent and recurrent CDI subsets.

For the economic outcomes in Table 2, all cost outcomes postdischarge among survivors were statistically similar between fidaxomicin and vancomycin in the overall cohort. These trends remained the same in the nonrecurrent and recurrent subsets. Moreover, in Table 1, hospital costs postindex within the index hospitalization for the overall cohort were also statistically similar.

Supplementary Tables 7 and 8 show the multivariable adjusted results from the multilevel hierarchical models with hospitals as random effects. Like the propensity-matched results, fidaxomicin was associated with a significantly higher sustained clinical response rate and lower CDI recurrence rate in the overall cohort and in the nonrecurrent and recurrent subsets. Fidaxomicin was associated with fewer CDI-related 30-, 60-, and 90-day readmissions compared with vancomycin in the overall cohort and in the nonrecurrent and recurrent subsets. Interestingly, in Supplementary Table 7, within the recurrent CDI patients, the costs postdischarge within 60 and 90 days were significantly lower in survivors who received fidaxomicin. For example, the 90-day adjusted mean total costs were $11 386 (95% CI, $9517–$13 255) for fidaxomicin patients and $14 238 (95% CI, $13 221–$15 256) for vancomycin patients (*P* = .023).

## DISCUSSION

We observed that the majority of patients with CDI were not receiving fidaxomicin treatment in accordance with the updated IDSA guidelines published in 2021. While fidaxomicin use for CDI has increased (and more than doubled) since the publication of the updated IDSA guidelines, it has remained low relative to vancomycin and metronidazole use. We also observed significant variations in the use of fidaxomicin by hospital characteristics, most notably by census region. These novel findings imply that a substantial number of patients could have received the benefits of fidaxomicin if more hospitals had followed the ISDA 2021 guidelines.

For the propensity-matched and multivariable regression results for the fidaxomicin vs vancomycin analysis, the outcomes of sustained clinical response and CDI recurrence are consistent with what has been observed in the literature. In the propensity-matched results, we observed a more favorable sustained response (OR, 1.48; 95% CI, 1.11–1.97) and a lower CDI recurrence rate associated with fidaxomicin (OR, 0.61; 95% CI, 0.44–0.85). Similarly, in a meta-analysis by Okumura et al., the observed sustained response was OR = 1.61 (95% CI, 1.27–2.05) and the CDI recurrence rate was OR = 0.50 (95% CI, 0.37–0.68) [14]. In a more recent meta-analysis, the CDI recurrence rate favoring fidaxomicin was relative risk = 0.59 (95% CI, 0.47–0.75) [16]. We did not see differences by treatment in the outcome of in-hospital mortality or discharge to hospice, but in select populations (eg, the nonrecurrent CDI cohort) there were differences in in-hospital mortality only. How the decision to admit patients to hospice is being made in the post period is beyond the study's scope, but our results do suggest that this is an important consideration when comparing short-term/in-hospital mortality in CDI patients.

Notably, fidaxomicin use significantly varied by census region and other hospital characteristics like number of beds. After controlling for hospital and patient characteristics, about a third of the variance in fidaxomicin use not explained by these factors was attributable to differences between hospitals. While those factors are unknown, hospital-level policies and physician attitudes toward fidaxomicin, particularly regarding its cost and third-party payer coverage, may affect usage. Vancomycin and metronidazole are markedly less expensive than fidaxomicin. Some hospital pharmacies will compound IV vancomycin into an oral solution to further save costs relative to fidaxomicin. However, research suggests that budgetary savings can be achieved due to reduced CDI recurrence and a superior sustained clinical response associated with fidaxomicin in the United States [22, 23]. Moreover, our findings consistently show that postindex costs were not statistically different between fidaxomicin and vancomycin either in the hospital or postdischarge within the first 90 days, and among patients with recurrent CDI fidaxomicin was associated with lower costs. Several smaller studies in the United Kingdom, France, and Germany also found that while fidaxomicin was associated with higher initial drug acquisition costs, first-line therapy of fidaxomicin was more cost-effective than vancomycin or metronidazole when examined over a longer time horizon due to its associated lower recurrence rate [24–27].

While our methodology and sample size are key strengths to our study, there are some limitations. The PINC AI Healthcare database is disproportionately represented by hospitals in the US South. How generalizable these findings are to populations outside the PINC AI Healthcare database is unknown, though the database represents ∼25% of US hospitals. Also, unmeasured confounding may be biasing our results, though our results are similar to other studies (including randomized controlled trials) in the literature. Finally, we stress that this study was done on a diverse group of hospitalized patients suffering from CDI. It is beyond the study's scope to compare guideline adherence and treatment outcomes of fidaxomicin with vancomycin or metronidazole within select subgroups of patients such as those older than 65, immunocompromised patients, or patients requiring intensive care. The limited research on select subgroups such as those who are immunocompromised or have a high level of acuity suggests that the benefits of fidaxomicin remain, though the relative differences in costs or treatment outcomes may vary [27–30].

## CONCLUSIONS

Since the 2021 guideline update, fidaxomicin use has increased significantly but remains underutilized. Notably, a majority of patients with CDI are not receiving treatment in accordance with the updated IDSA guidelines. Consistent with prior research, we found that fidaxomicin was associated with better sustained clinical response and lower CDI recurrence compared with vancomycin in a large, modern multicenter analysis. Furthermore, we observed that postdischarge costs associated with fidaxomicin were either lower than costs associated with vancomycin use or not significantly different. Another novel finding was that fidaxomicin use varied by census region and other hospital characteristics. We believe these results further demonstrate the real-world value of fidaxomicin and may be used to better target adherence to the updated IDSA guidelines for CDI.

## Supplementary Material

ofae524_Supplementary_Data

## Supplementary Data

Supplementary materials are available at *Open Forum Infectious Diseases* online. Consisting of data provided by the authors to benefit the reader, the posted materials are not copyedited and are the sole responsibility of the authors, so questions or comments should be addressed to the corresponding author.
