# Supplementary material for: A Retrospective Assessment of Guideline Adherence and Treatment Outcomes From Clostridioides difficile Infection Following the IDSA 2021 Clinical Guideline Update: Clostridioides difficile Infection
Source: Open Forum Infect Dis. 2024 Sep 30;11(10):ofae524. doi: 10.1093/ofid/ofae524 (PMC11443340; doi:10.1093/ofid/ofae524)
Supplement: ofae524_Supplementary_Data [file ofae524_supplementary_data.docx]

Supplemental Table 1: Clinical Outcome Definitions

| Variable | Definition |
| --- | --- |
| Clinical Resolution | - If length of index treatment <10 days: discharged alive while still on treatment and no hospital readmission with CDI as the primary diagnosis within (10 - length of the index CDI treatment) days of the index discharge - If length of index treatment ≥ 10 days: the index treatment continued until discharge: discharge alive and no hospital readmissions with CDI as the primary diagnosis within 1 day of the index discharge - If the index treatment ended before discharge: patient was alive without any CDI treatment for at least one day after the end of index treatment |
| CDI recurrence | - CDI recurrence is reported only in those patients experiencing clinical resolution. - CDI Recurrence is defined as any evidence of a new CDI treatment after the index treatment or hospitalization with a CDI diagnosis (a CDI diagnosis + CDI treatment) within 30 days (of the date of completion of index CDI prescription in those patients with clinical resolution. |
| Sustained clinical response | Sustained response was defined as a combination of the following:   - Clinical resolution combined with: - No CDI recurrence defined as the absence of the evidence of hospitalization with a CDI as the primary diagnosis within 30 days of the date of completion of index CDI prescription |

Supplemental Table 2: High Risk Antibiotics

| Amox/Pot Clav | Dicloxacillin |
| --- | --- |
| Amoxicillin | Dirithromycin |
| Ampicillin | Doripenem |
| Ampicillin/Sulbac | Enoxacin |
| Azithromycin | Ertapenem |
| Bacampicillin | Erythro Base |
| Carbenicillin | Erythro Ethyl |
| Cefaclor | Erythro Lacto |
| Cefadroxil | Erythro/Sulfisox |
| Cefamandole | Fidaxomicin |
| Cefazolin | Gatifloxacin |
| Cefdinir | Gemifloxacin |
| Cefditoren Pivoxil | Imipenem |
| Cefepime | Imipenem/Cilastin/Relebactam |
| Cefiderocol | Levofloxacin |
| Cefixime | Lomefloxacin |
| Cefonicid | Loracarbef |
| Cefoperazone | Meropenem |
| Cefotaxime | Meropenem/Vaborbactam |
| Cefotetan | Methicillin |
| Cefoxitin | Mezlocillin |
| Cefpodoxime | Moxifloxacin |
| Cefprozil | Nafcillin |
| Ceftaroline | Nalidixic Acid |
| Ceftazidime | Norfloxacin |
| Ceftazidime/ Avibactam | Ofloxacin |
| Ceftazidime/Avibactam | Oxacillin |
| Ceftibuten | Pcn G Benz |
| Ceftizoxime | Pcn G Benz/Proc |
| Ceftolozane/Tazobacam | Pcn G Na |
| Ceftriaxone | Pcn G Pot |
| Cefuroxime | Pcn G Proc |
| Cephalexin | Pcn V Pot |
| Cephalothin | Piperacillin |
| Cephapirin | Piperacillin/Tazo |
| Cephradine | Sparfloxacin |
| Cinoxacin | Telithromycin |
| Ciprofloxacin | Ticar/Clav Pot |
| Clarithromycin | Ticarcillin |
| Clindamycin | Troleandomycin |
| Cloxacillin | Trovafloxacin |
| Delafloxacin |  |

Supplemental Table 3: Overall Baseline characteristics for Patients in Study Sample Pre vs Post 2021 Guideline Update

| **Patient Characteristics** | **Pre-Guidelines, N = 29520** | **%** | **Post-Guidelines N = 15529** | **%** | **P-value** | |  |  |
| --- | --- | --- | --- | --- | --- | --- | --- | --- |
| Age, years (Mean (SD)) | 66.8 (15.9) |  | 67.5 (15.6) |  | <0.001 | | | |
| Gender: male | 12061 | 40.86% | 6429 | 41.40% | 0.266 | | | |
| **Race** |  |  |  |  |  | | | |
| White | 23668 | 80.18% | 12594 | 81.10% |  | | | |
| Black | 3552 | 12.03% | 1781 | 11.47% |  | | | |
| Asian | 443 | 1.50% | 272 | 1.75% | 0.003 | | | |
| Other | 1417 | 4.80% | 653 | 4.21% |  | | | |
| Unknown | 440 | 1.49% | 229 | 1.47% |  | | | |
| Hispanic | 2026 | 6.86% | 1323 | 8.52% | <0.001 | | | |
| **Admission Source for Hospitalized Patients** |  |  |  |  |  | | | |
| Non-healthcare facility (including from home) | 24486 | 82.95% | 13164 | 84.77% |  | | | |
| Clinic | 2006 | 6.80% | 664 | 4.28% |  | | | |
| Transfer from a different hospital | 1300 | 4.40% | 633 | 4.08% | <0.001 | | | |
| Transfer from a SNF/ICF/Different Health Facility | 1315 | 4.45% | 795 | 5.12% |  | | | |
| Other | 413 | 1.40% | 273 | 1.76% |  | | | |
| **Insurance Type** |  |  |  |  |  | | | |
| Medicare | 20433 | 69.22% | 10877 | 70.04% |  | | | |
| Medicaid | 3697 | 12.52% | 1920 | 12.36% |  | | | |
| Managed Care | 2872 | 9.73% | 1460 | 9.40% | 0.006 | | | |
| Commercial/Workers Compensation | 1310 | 4.44% | 668 | 4.30% |  | | | |
| Self-Pay | 457 | 1.55% | 177 | 1.14% |  | | | |
| Other | 751 | 2.54% | 427 | 2.75% |  | | | |
| **Surgical vs Medical (when available for hospitalized patients)** |  |  |  |  |  | |  |  |
| Surgical | 3715 | 12.58% | 1897 | 12.22% | 0.260 | |  |  |
| Medical | 25805 | 87.42% | 13632 | 87.78% |  | | | |
| **Admission Type (for hospitalized patients)** |  |  |  |  |  | | | |
| Emergency | 24981 | 84.62% | 13447 | 86.59% |  | | | |
| Urgent | 3390 | 11.48% | 1538 | 9.90% | <0.001 | | | |
| Elective/Other | 1149 | 3.89% | 544 | 3.50% |  | | | |
| **Elixhauser Comorbidities** |  |  |  |  |  | |  |  |
| Congestive heart failure | 8614 | 29.18% | 4658 | 30.00% | 0.071 | | | |
| Cardiac Arrhythmias | 10134 | 34.33% | 5511 | 35.49% | 0.014 | | | |
| Valvular disease | 2859 | 9.68% | 1536 | 9.89% | 0.483 | | | |
| Pulmonary circulation disease | 2169 | 7.35% | 1174 | 7.56% | 0.413 | | | |
| Peripheral vascular disease | 3435 | 11.64% | 1822 | 11.73% | 0.761 | | | |
| Paralysis | 634 | 2.15% | 350 | 2.25% | 0.464 | | | |
| Other neurological disorders | 6649 | 22.52% | 3633 | 23.39% | 0.036 | | | |
| Chronic pulmonary disease | 8166 | 27.66% | 4247 | 27.35% | 0.479 | | | |
| Diabetes without chronic complications | 2256 | 7.64% | 1210 | 7.79% | 0.571 | | | |
| Diabetes with chronic complications | 9073 | 30.74% | 4888 | 31.48% | 0.106 | | | |
| Hypothyroidism | 5885 | 19.94% | 3167 | 20.39% | 0.248 | | | |
| Renal failure | 11218 | 38.00% | 5853 | 37.69% | 0.518 | | | |
| Liver disease | 3416 | 11.57% | 1835 | 11.82% | 0.442 | | | |
| Peptic ulcer disease with bleeding | 407 | 1.38% | 199 | 1.28% | 0.394 | | | |
| AIDS | 153 | 0.52% | 85 | 0.55% | 0.686 | | | |
| Lymphoma | 804 | 2.72% | 465 | 2.99% | 0.099 | | | |
| Metastatic cancer | 1723 | 5.84% | 901 | 5.80% | 0.881 | | | |
| Solid tumor without metastasis | 3256 | 11.03% | 1728 | 11.13% | 0.753 | | | |
| Rheumatoid arthritis/collagen vascular | 1869 | 6.33% | 941 | 6.06% | 0.257 | | | |
| Coagulopathy | 3896 | 13.20% | 2169 | 13.97% | 0.023 | | | |
| Obesity | 5496 | 18.62% | 2959 | 19.05% | 0.259 | | | |
| Weight loss | 6276 | 21.26% | 3228 | 20.79% | 0.242 | | | |
| Fluid and electrolyte disorders | 21568 | 73.06% | 11336 | 73.00% | 0.885 | | | |
| Chronic blood loss anemia | 483 | 1.64% | 239 | 1.54% | 0.435 | | | |
| Deficiency anemia | 2813 | 9.53% | 1574 | 10.14% | 0.039 | | | |
| Alcohol abuse | 2328 | 7.89% | 1130 | 7.28% | 0.021 | | | |
| Drug abuse | 1604 | 5.43% | 799 | 5.15% | 0.195 | | | |
| Psychosis | 454 | 1.54% | 241 | 1.55% | 0.909 | | | |
| Depression | 6349 | 21.51% | 3304 | 21.28% | 0.570 | | | |
| Hypertension | 22477 | 76.14% | 12088 | 77.84% | <0.001 | | | |
| **Charlson Comorbidity Score** |  |  |  |  |  | |  |  |
| Mean (SD) | 3.3 (2.5) |  | 3.3 (2.5) |  | 0.665 | | | |
| Median [IQR] | 3 [1, 5] |  | 3 [1, 5] |  | 0.481 | | | |
| **Additional Comorbidities** |  |  |  |  |  | | | |
| Crohn’s disease or ulcerative colitis | 1675 | 5.67% | 921 | 5.93% | 0.266 | | | |
| Solid organ transplant | 484 | 1.64% | 276 | 1.78% | 0.281 | | | |
| Bone Marrow/Stem Cell Transplant | 246 | 0.83% | 141 | 0.91% | 0.415 | | | |
| **Sepsis Present on Admission** | 9478 | 32.11% | 5160 | 33.23% | 0.016 | | | |
| **Antibiotics On or Before First Day of Treatment** |  |  |  |  |  | | | |
| Moderate to high-risk antibiotics | 20138 | 68.22% | 10822 | 69.69% | 0.001 | | | |
| Low risk antibiotics | 2344 | 7.94% | 1369 | 8.82% | 0.001 | | | |
| **Antibiotics On First Day of Treatment** |  |  |  |  |  | | | |
| Moderate to high-risk antibiotics | 15540 | 52.64% | 8178 | 52.66% | 0.967 | | | |
| Low risk antibiotics | 1632 | 5.53% | 910 | 5.86% | 0.147 | | | |
| **Additional Treatments of interest On or Before First Day of Treatment** |  |  |  |  |  | | | |
| Bezlotoxumab | 0 | 0.00% | 8 | 0.05% | <0.001 | | | |
| Tigecyclcine | 75 | 0.25% | 44 | 0.28% | 0.565 | | | |
| Rifaximin | 168 | 0.57% | 105 | 0.68% | 0.164 | | | |
| Nitazoxanide | 18 | 0.06% | 8 | 0.05% | 0.691 | | | |
| Fecal Microbiota Transplant | 3 | 0.01% | 2 | 0.01% | 1.000 | | |  |
| **Colectomy post first day treatment** | 1 | 0.00% | 1 | 0.01% | 1.000 | | | |
| **Additional Treatments of interest (ANY DAY)** |  |  |  |  |  | | | |
| Bezlotoxumab | 10 | 0.03% | 34 | 0.22% | <0.001 | | | |
| Tigecyclcine | 159 | 0.54% | 94 | 0.61% | 0.368 | | | |
| Rifaximin | 290 | 0.98% | 151 | 0.97% | 0.918 | | | |
| Nitazoxanide | 33 | 0.11% | 12 | 0.08% | 0.270 | | | |
| Fecal Microbiota Transplant | 19 | 0.06% | 7 | 0.05% | 0.418 | | | |
| Colectomy | 4 | 0.01% | 1 | 0.01% | 0.666 | | | |
| **Acuity Measures (for Hospitalized Patients) Before First Day of Treatment** |  |  |  |  |  | | | |
| ICU Admission | 3790 | 12.84% | 1905 | 12.27% | 0.083 | | | |
| Mechanical Ventilation | 1275 | 4.32% | 617 | 3.97% | 0.082 | | | |
| Dialysis | 1857 | 6.29% | 1027 | 6.61% | 0.183 | | | |
| H2 Blocker | 3592 | 12.17% | 1831 | 11.79% | 0.242 | | | |
| Proton Pump Inhibitors | 8358 | 28.31% | 4782 | 30.79% | <0.001 | | | |
| **Acuity Measures (for Hospitalized Patients) On or Before First Day of Treatment** |  |  |  |  |  | |  |  |
| ICU Admission | 5116 | 17.33% | 2479 | 15.96% | <0.001 | | | |
| Mechanical Ventilation | 1572 | 5.33% | 748 | 4.82% | 0.020 | | | |
| Dialysis | 2602 | 8.81% | 1345 | 8.66% | 0.585 | | | |
| H2 Blocker | 5405 | 18.31% | 2686 | 17.30% | 0.008 | | | |
| Proton Pump Inhibitors | 12621 | 42.75% | 6914 | 44.52% | <0.001 | | | |
| **Hospital Characteristics** |  |  |  |  |  |  |  |  |
| **Census region** |  |  |  |  |  |  |  |  |
| Midwest | 8118 | 27.50% | 4188 | 26.97% |  |  |  |  |
| Northeast | 4367 | 14.79% | 2104 | 13.55% | <0.001 |  |  |  |
| South | 12192 | 41.30% | 6161 | 39.67% |  |  |  |  |
| West | 4843 | 16.41% | 3076 | 19.81% |  |  |  |  |
| **Number of Beds** |  |  |  |  |  |  |  |  |
| <200 | 6391 | 21.65% | 3144 | 20.25% |  |  |  |  |
| 200 to 399 | 10042 | 34.02% | 5697 | 36.69% | <0.001 |  |  |  |
| 400+ | 13087 | 44.33% | 6688 | 43.07% |  |  |  |  |
| **Teaching** | 13942 | 47.23% | 7062 | 45.48% | <0.001 |  |  |  |
| **Urban** | 25145 | 85.18% | 13207 | 85.05% | 0.708 |  |  |  |

Supplement Table 4: Attrition Table

| **Inclusion Criteria** | **Sample Size Remaining after the criterion is applied** |
| --- | --- |
| Inpatient admitted from 1/2020 to 9/2022 | 18,128,089 |
| Patients Age 18 or older | 15,669,244 |
| Patients with an ICD-10 code of A04.7x | 124,720 |
| Treatment of Oral Vancomycin, Oral Fidaxomicin, Oral or IV Metronidazole for at least 1 day | 111,993 |
| Treatment of Oral Vancomycin, Oral Fidaxomicin, Oral or IV Metronidazole for at least 10 consecutive days or at least 1 of the above drugs given on the last day of hospitalization | 93,916 |
| **Exclusion Criteria** |  |
| Missing Gender | 93,832 |
| Missing Patient Costs or Patient Costs = $0 | 93,557 |
| Still a Patient or unknown Discharge Status Location | 93,488 |
| Laxatives at any time | 80,004 |
| Exclude patients in “Wash-Out”  period p (7/2021 to 9/2021) | 72,339 |
| Exclude multiple encounters--only examine the first encounter during the study period. | 63,506 |
| Exclude patients without at least one record in PHD within 365 days prior to the index admission | **45,049; sample size for treatment patterns** |
| *Exclude patients with fidaxomicin treatment (10+ days or treated on the last day) treated with at least 1 day of vancomycin or metronidazole and vancomycin treatment (10+ days or treated on the last day) treated with at least 1 day of fidaxomicin or metronidazole* | ***24,853; sample size for the comparison between fidaxomicin and vancomycin*** |

Supplemental Table 5: Additional Patient Characteristics and Clinical Outcomes

| **Characteristics** | **Pre-Guidelines, N = 29520** | **%** | **Post-Guidelines N = 15529** | **%** | **P-value** |
| --- | --- | --- | --- | --- | --- |
| **C Diff Drug given for at least 1 day** |  |  |  |  |  |
| Vancomycin | 27512 | 93.20% | 14054 | 90.50% | <0.001 |
| Metronidazole | 13000 | 44.04% | 5887 | 37.91% | <0.001 |
| Fidaxomicin | 1993 | 6.75% | 2409 | 15.51% | <0.001 |
| **C Diff Drug given for 10 or More Days or on the Last Hospital Day** |  |  |  |  |  |
| Vancomycin | 25956 | 87.93% | 12877 | 82.92% | <0.001 |
| Metronidazole | 6365 | 21.56% | 2678 | 17.25% | <0.001 |
| Fidaxomicin | 1748 | 5.92% | 2126 | 13.69% | <0.001 |
| Non-Recurrent CDI | 25267 | 85.59% | 13446 | 86.59% |  |
| Recurrent CDI | 4253 | 14.41% | 2083 | 13.41% | 0.004 |
| **Index Day (Mean (SD))** | 3.5 (4.7) |  | 3.7 (4.6) |  | 0.002 |
| **Index Day (Median [IQR])** | 2 [1, 4] |  | 2 [1, 4] |  | <0.001 |
| **Outcomes** |  |  |  |  |  |
| Clinical Resolution | 28386 | 96.16% | 14897 | 95.93% | 0.235 |
| Sustained Clinical Response | 25350 | 85.87% | 13416 | 86.39% | 0.131 |
| CDI Recurrence | 3036 | 10.70% | 1481 | 9.94% | 0.015 |
| In-Hospital Mortality | 1162 | 3.94% | 712 | 4.58% | 0.001 |
| In-Hospital Mortality or Discharged to Hospice | 2506 | 8.49% | 1312 | 8.45% | 0.884 |
| Total Length of Stay | 8.5 (9.0) |  | 8.9 (9.8) |  | <0.001 |
| All Cause Readmission within 30 Days | 5792 | 20.42% | 2825 | 19.07% | 0.001 |
| All Cause Readmission within 60 Days | 8225 | 29.00% | 3855 | 26.02% | <0.001 |
| All Cause Readmission within 90 Days | 9521 | 33.57% | 4364 | 29.45% | <0.001 |
| CDI-Related Readmission within 30 Days | 2877 | 10.15% | 1336 | 9.02% | <0.001 |
| CDI-Related Readmission within 60 Days | 3789 | 13.36% | 1705 | 11.51% | <0.001 |
| CDI-Related Readmission within 90 Days | 4185 | 14.76% | 1857 | 12.53% | <0.001 |
| ER Visits within 30 Days | 3039 | 10.72% | 1710 | 11.54% | 0.009 |
| ER Visits within 60 Days | 4532 | 15.98% | 2447 | 16.51% | 0.153 |
| ER Visits within 90 Days | 5545 | 19.55% | 2900 | 19.57% | 0.963 |
| CDI-Related ER visits within 30 Days | 394 | 1.39% | 202 | 1.36% | 0.825 |
| CDI-Related ER visits within 60 Days | 504 | 1.78% | 251 | 1.69% | 0.531 |
| CDI-Related ER visits within 90 Days | 560 | 1.97% | 274 | 1.85% | 0.368 |
| Total Hospital Costs (Mean (SD)) | 23084 (35813) |  | 24572 (35404) |  | <0.001 |
| Post-Index LOS (Mean (SD)) | 5.9 (6.8) |  | 6.2 (7.8) |  | <0.001 |
| Hospital Costs Post Index (Mean (SD)) | 14734 (25303) |  | 15504 (24592) |  | 0.002 |
| All Costs Post Discharge within 30 Days for survivors; No Hospice patients (Mean (SD) | 5877 (17876) |  | 5867 (17706) |  | 0.960 |
| All Costs Post Discharge within 60 Days for survivors; No Hospice patients (Mean (SD)) | 9560 (30902) |  | 8851 (22088) |  | 0.015 |
| All Costs Post Discharge within 90 Days for survivors; No Hospice patients (Mean (SD)) | 12431 (34554) |  | 11060 (26941) |  | <0.001 |

Supplemental Table 6: Number of Matched Pairs After Propensity Matching

| Group | **All Patients, Outcomes:**  Clinical Resolution;  Sustained Clinical Response;  CDI Recurrence;  In-Hospital Mortality;  In-Hospital Mortality or Discharged to Hospice;  Total Length of Stay;  Total Hospital Costs;  Post-Index LOS;  Hospital Costs Post Index;  Hospital Charges Post Index | **Survived to Discharge, Outcomes**:  All Cause Readmission within 30, 60, and 90 Days;  CDI-Related Readmission within 30, 60, and 90 Days;  ER Visits within 30, 60, and 90 Days;  CDI-Related ER visits within 30, 60, and 90 Days | **Survived to Discharge, not admitted to Hospice Outcomes:**  All Costs Post Discharge within 30, 60, and 90 Days for survivors;  All Charges Post Discharge within 30,60, and 90 Days for survivors |
| --- | --- | --- | --- |
| Overall | 1,062 out of 1,075 | 1,030 out of 1,051 | 1,009 out of 1,020 |
| Non-Recurrent | 625 out of 626 | 600 out of 605 | 584 out of 587 |
| Recurrent | 422 out of 449 | 419 out of 446 | 411 out of 433 |

Supplemental Table 7: Adjusted Clinical Outcome Results for Fidaxomicin vs. Vancomycin comparison from Multivariable Hierarchical Modeling

| **Overall** | **Vancomycin: Rate or Mean; 95% Confidence Interval** | **Fidaxomicin: Rate or Mean; 95% Confidence Interval** | **P-value** |
| --- | --- | --- | --- |
| Clinical Resolution | 97.72%; (97.52%, 97.93%) | 97.53%; (96.52%, 98.53%) | 0.700 |
| Sustained Clinical Response | 87.80%; (87.34%, 88.25%) | 91.67%; (89.99%, 93.35%) | <0.001 |
| CDI Recurrence | 10.18%; (9.76%, 10.60%) | 6.13%; (4.68%, 7.59%) | <0.001 |
| In-Hospital Mortality | 2.17%; (1.98%, 2.36%) | 2.85%; (1.80%, 3.91%) | 0.161 |
| In-Hospital Mortality or Discharged to Hospice | 5.62%; (5.31%, 5.93%) | 6.00%; (4.54%, 7.45%) | 0.611 |
| Total Length of Stay | 13.68; (12.27, 15.09) | 14.51; (12.92, 16.09) | 0.002 |
| All Cause Readmission within 30 Days | 20.73%; (20.19%, 21.28%) | 17.02%; (14.71%, 19.33%) | 0.004 |
| All Cause Readmission within 60 Days | 28.92%; (28.30%, 29.53%) | 26.59%; (23.89%, 29.29%) | 0.107 |
| All Cause Readmission within 90 Days | 33.19%; (32.53%, 33.86%) | 30.73%; (27.91%, 33.55%) | 0.100 |
| CDI-Related Readmission within 30 Days | 9.87%; (9.47%, 10.27%) | 6.27%; (4.80%, 7.74%) | <0.001 |
| CDI-Related Readmission within 60 Days | 12.69%; (12.25%, 13.14%) | 8.76%; (7.07%, 10.44%) | <0.001 |
| CDI-Related Readmission within 90 Days | 13.94%; (13.46%, 14.43%) | 10.26%; (8.45%, 12.06%) | 0.001 |
| ER Visits within 30 Days | 11.67%; (11.10%, 12.23%) | 9.80%; (7.91%, 11.69%) | 0.075 |
| ER Visits within 60 Days | 17.05%; (16.33%, 17.77%) | 14.71%; (12.48%, 16.94%) | 0.053 |
| ER Visits within 90 Days | 20.52%; (19.74%, 21.29%) | 17.21%; (14.85%, 19.57%) | 0.010 |
| CDI-Related ER visits within 30 Days | 1.36%; (1.20%, 1.52%) | 1.25%; (0.58%, 1.92%) | 0.754 |
| CDI-Related ER visits within 60 Days | 1.71%; (1.53%, 1.88%) | 1.66%; (0.91%, 2.40%) | 0.900 |
| CDI-Related ER visits within 90 Days | 1.84%; (1.66%, 2.03%) | 1.88%; (1.09%, 2.67%) | 0.930 |
| Total Hospital Costs | $27,035; ($25,528, $28,543) | $31,159 ; ($29,074, $33,244) | <0.001 |
| Post-Index LOS | 5.02; (4.88, 5.16) | 5.14; (4.8, 5.48) | 0.456 |
| Hospital Costs Post Index | $12,266; ($11,795, $12,737) | $15,499; ($14,462, $16,536) | <0.001 |
| Hospital Charges Post Index | $43,074; ($40,869, $45,280) | $56,913; ($52,702, $61,123) | <0.001 |
| **Non-Recurrent** | **Vancomycin: Rate or Mean; 95% Confidence Interval** | **Fidaxomicin: Rate or Mean; 95% Confidence Interval** | **P-value** |
| Clinical Resolution | 97.63%; (97.42%, 97.85%) | 97.46%; (96.23%, 98.70%) | 0.783 |
| Sustained Clinical Response | 88.03%; (87.55%, 88.50%) | 91.52%; (89.30%, 93.74%) | 0.009 |
| CDI Recurrence | 9.85%; (9.42%, 10.29%) | 6.14%; (4.19%, 8.09%) | 0.003 |
| In-Hospital Mortality | 2.23%; (2.03%, 2.42%) | 3.73%; (2.28%, 5.17%) | 0.012 |
| In-Hospital Mortality or Discharged to Hospice | 5.77%; (5.44%, 6.10%) | 6.76%; (4.86%, 8.65%) | 0.283 |
| Total Length of Stay | 14.26; (12.71, 15.81) | 15.55; (13.71, 17.38) | <0.001 |
| All Cause Readmission within 30 Days | 20.55%; (19.97%, 21.12%) | 17.37%; (14.34%, 20.40%) | 0.057 |
| All Cause Readmission within 60 Days | 28.43%; (27.77%, 29.09%) | 26.96%; (23.42%, 30.50%) | 0.429 |
| All Cause Readmission within 90 Days | 32.58%; (31.88%, 33.29%) | 31.16%; (27.47%, 34.84%) | 0.459 |
| CDI-Related Readmission within 30 Days | 9.64%; (9.22%, 10.06%) | 5.98%; (4.06%, 7.91%) | 0.003 |
| CDI-Related Readmission within 60 Days | 12.16%; (11.70%, 12.63%) | 7.92%; (5.72%, 10.13%) | 0.002 |
| CDI-Related Readmission within 90 Days | 13.25%; (12.76%, 13.75%) | 9.77%; (7.35%, 12.19%) | 0.015 |
| ER Visits within 30 Days | 11.70%; (11.11%, 12.29%) | 10.28%; (7.80%, 12.79%) | 0.29 |
| ER Visits within 60 Days | 16.92%; (16.19%, 17.66%) | 15.08%; (12.17%, 18.00%) | 0.238 |
| ER Visits within 90 Days | 20.25%; (19.47%, 21.03%) | 16.79%; (13.76%, 19.81%) | 0.037 |
| CDI-Related ER visits within 30 Days | 1.32%; (1.16%, 1.48%) | 1.06%; (0.22%, 1.90%) | 0.586 |
| CDI-Related ER visits within 60 Days | 1.61%; (1.43%, 1.78%) | 1.39%; (0.43%, 2.34%) | 0.677 |
| CDI-Related ER visits within 90 Days | 1.72%; (1.54%, 1.90%) | 1.56%; (0.55%, 2.58%) | 0.777 |
| Total Hospital Costs | $28,057; ($26,387 , $29,727) | $33,496; ($30,951 , $36,040) | <0.001 |
| Post-Index LOS | 4.98; (4.84, 5.12) | 4.98; (4.57, 5.39) | 0.988 |
| Hospital Costs Post Index | $12,223; ($11,747, $12699) | $16,014; ($14,715 , $17,313) | <0.001 |
| Hospital Charges Post Index | $42,773; ($40,552, $44,995) | $58,490; ($53,411 , $63,569) | <0.001 |
| **Recurrent** | **Vancomycin: Rate or Mean; 95% Confidence Interval** | **Fidaxomicin: Rate or Mean; 95% Confidence Interval** | **P-value** |
| Clinical Resolution | 98.09%; (97.58%, 98.60%) | 97.83%; (96.25%, 99.40%) | 0.749 |
| Sustained Clinical Response | 86.16%; (84.91%, 87.41%) | 90.82%; (88.03%, 93.61%) | 0.010 |
| CDI Recurrence | 12.43%; (11.22%, 13.64%) | 7.44%; (4.91%, 9.98%) | 0.004 |
| In-Hospital Mortality | 2.07%; (1.56%, 2.57%) | 0.72%; (0.00%, 1.60%) | 0.094 |
| In-Hospital Mortality or Discharged to Hospice | 4.83%; (4.07%, 5.59%) | 4.47%; (2.36%, 6.59%) | 0.767 |
| Total Length of Stay | 7.71; (7.08, 8.35) | 7.94; (7.13, 8.75) | 0.399 |
| All Cause Readmission within 30 Days | 22.05%; (20.53%, 23.58%) | 17.40%; (13.80%, 21.00%) | 0.030 |
| All Cause Readmission within 60 Days | 32.25%; (30.50%, 34.01%) | 27.58%; (23.37%, 31.78%) | 0.052 |
| All Cause Readmission within 90 Days | 37.41%; (35.62%, 39.20%) | 32.52%; (28.12%, 36.91%) | 0.050 |
| CDI-Related Readmission within 30 Days | 11.39%; (10.22%, 12.56%) | 7.59%; (5.07%, 10.11%) | 0.021 |
| CDI-Related Readmission within 60 Days | 16.32%; (14.96%, 17.69%) | 11.91%; (8.86%, 14.96%) | 0.020 |
| CDI-Related Readmission within 90 Days | 18.77%; (17.33%, 20.21%) | 13.79%; (10.54%, 17.05%) | 0.013 |
| ER Visits within 30 Days | 11.34%; (10.11%, 12.57%) | 9.41%; (6.58%, 12.25%) | 0.248 |
| ER Visits within 60 Days | 17.71%; (16.16%, 19.25%) | 15.19%; (11.74%, 18.65%) | 0.206 |
| ER Visits within 90 Days | 21.82%; (20.15%, 23.50%) | 18.97%; (15.23%, 22.70%) | 0.181 |
| CDI-Related ER visits within 30 Days | 2.09%; (1.45%, 2.74%) | 2.88%; (0.87%, 4.89%) | 0.420 |
| CDI-Related ER visits within 60 Days | 2.87%; (2.15%, 3.59%) | 3.64%; (1.57%, 5.72%) | 0.458 |
| CDI-Related ER visits within 90 Days | 3.11%; (2.37%, 3.86%) | 4.00%; (1.88%, 6.12%) | 0.400 |
| Total Hospital Costs | $19,532; ($18,053, $21,011) | $21,431; ($19,326, $23,536) | 0.003 |
| Post-Index LOS | 4.95; (4.66, 5.24) | 4.81; (4.23, 5.39) | 0.638 |
| Hospital Costs Post Index | $12,668; ($12,014, $13,321) | $14,574; ($13,175, $15,973) | 0.008 |
| Hospital Charges Post Index | $45,238; ($42,370, $48,105) | $55,065; ($49,389, $60,742) | <0.001 |

Supplemental Table 8: Economic Outcome Results for Fidaxomicin vs. Vancomycin comparison from Multivariable Hierarchical Modeling

| Overall Cohort | Vancomycin: Mean;  95% Confidence Interval | Fidaxomicin: Mean;  95% Confidence Interval | P-value |
| --- | --- | --- | --- |
| All Costs Post Discharge within 30 Days for survivors; No Hospice patients | $6,064;($5,779,$6,349) | $5,523;($4,595,$6,452) | 0.413 |
| All Costs Post Discharge within 60 Days for survivors; No Hospice patients | $9,577;($9,144,$10,009) | $8,937;($7,736,$10,138) | 0.346 |
| All Costs Post Discharge within 90 Days for survivors; No Hospice patients | $12,334;($11,775,$12,893) | $11,218;($9,815,$12,620) | 0.085 |
| Non-Recurrent Cohort | Vancomycin: Mean;  95% Confidence Interval | Fidaxomicin: Mean;  95% Confidence Interval | P-value |
| All Costs Post Discharge within 30 Days for survivors; No Hospice patients | $6,065;($5,757,$6,373) | $5,831;($4,442,$7,219) | 0.823 |
| All Costs Post Discharge within 60 Days for survivors; No Hospice patients | $9,378;($8,935,$9,821) | $9,681;($7,790,$11,571) | 0.827 |
| All Costs Post Discharge within 90 Days for survivors; No Hospice patients | $12,041;($11,462,$12,619) | $11,916;($9,816,$14,015) | 0.520 |
| Recurrent Cohort | Vancomycin: Mean;  95% Confidence Interval | Fidaxomicin: Mean;  95% Confidence Interval | P-value |
| All Costs Post Discharge within 30 Days for survivors; No Hospice patients | $6,037;($5,513,$6,562) | $5,210;($4,003,$6,418) | 0.312 |
| All Costs Post Discharge within 60 Days for survivors; No Hospice patients | $10,807;($9,968,$11,645) | $8,815;($7,270,$10,359) | **0.039** |
| All Costs Post Discharge within 90 Days for survivors; No Hospice patients | $14,238;($13,221,$15,256) | $11,386;($9,517,$13,255) | **0.023** |
